# Limited Role of *Mycoplasma genitalium, Chlamydia trachomatis*, and *Neisseria gonorrhoeae* in Pelvic Inflammatory Disease and Tubo‐Ovarian Abscess in the Netherlands: A Multicenter Prospective Cohort Study

**DOI:** 10.1155/ogi/3810643

**Published:** 2026-09-12

**Authors:** Nikki Adriaens, Sylvia M. Bruisten, Clarissa E. Vergunst, Vita W. Jongen, Joyce F. Braam, Roel H. T. Nijhuis, Arien Bukman, Paul J. M. van Kesteren, Paul Gruteke, Merel van Doorenmaalen, Alje P. van Dam

**Affiliations:** ^1^ Department of Infectious Diseases, Public Health Service of Amsterdam, Amsterdam, the Netherlands, ggd.amsterdam.nl; ^2^ Infectious Diseases, Amsterdam Institute for Immunology and Infectious Diseases, Amsterdam, the Netherlands; ^3^ Department of Dermatology, NWZ, Den Helder, the Netherlands; ^4^ Stichting HIV Monitoring, Amsterdam, the Netherlands; ^5^ Department of Medical Microbiology & Medical Immunology, Meander Medical Centre, Amersfoort, the Netherlands; ^6^ Department of Obstetrics & Gynecology, Meander Medical Centre, Amersfoort, the Netherlands; ^7^ Department of Obstetrics & Gynecology and Medical Microbiology, OLVG, Amsterdam, the Netherlands; ^8^ Department of Medical Microbiology, OLVG Laboratory, Amsterdam, the Netherlands; ^9^ Department of Obstetrics & Gynecology, Flevo Hospital, Almere, the Netherlands, flevoziekenhuis.nl; ^10^ Department of Medical Microbiology, Amsterdam UMC Location, University of Amsterdam, Amsterdam, the Netherlands

**Keywords:** antibiotic treatment, *Chlamydia trachomatis*, *Mycoplasma genitalium*, *Neisseria gonorrhoeae*, pelvic inflammatory disease, sexually transmitted infection, the Netherlands, tubo-ovarian abscess

## Abstract

**Objective:**

To evaluate the role of *Mycoplasma genitalium*, *Chlamydia trachomatis*, and *Neisseria gonorrhoeae* in pelvic inflammatory disease (PID) and tubo‐ovarian abscess (TOA), and to assess the effectiveness of standard therapy in achieving clinical and microbiological cure.

**Design:**

Multicenter prospective cohort study.

**Setting:**

Three hospitals and one Centre for Sexual Health (CSH) in the Netherlands.

**Population:**

Women ≥ 18 years with clinically diagnosed PID and/or TOA between 2021 and 2023.

**Methods:**

Clinical examination, C‐reactive protein (CRP) levels, and vaginal swabs were evaluated at baseline and follow‐up. Participants completed a self‐reported symptom diary. Antibiotic therapy was prescribed in accordance with Dutch national hospital and CSH guidelines.

**Main Outcome Measures:**

The primary outcome, clinical cure at Week 4, was defined as the absence of fever and vomiting, CRP < 10 mg/L, and ≥ 70% reduction in symptom score. Microbiological cure was the secondary outcome.

**Results:**

Among 100 women with PID and/or TOA, monoinfections with *M. genitalium, C. trachomatis*, and *N. gonorrhoeae* were detected in 4.0%, 6.0%, and 9.0% of cases, respectively; coinfections occurred in 4.0%, while 77.0% tested negative for all three pathogens. By Week 4, *M. genitalium* and *C. trachomatis* were cleared, with one case of persistent or recurrent *N. gonorrhoeae*. Symptom scores declined by 91.3%, with significant CRP reduction. Clinical cure was achieved in 67.3% of participants, with no association between cure and sexually transmitted infection (STI) status or diagnosis.

**Conclusions:**

Most PID and/or TOA cases lacked an identifiable STI etiology. Nonetheless, the majority showed clinical and microbiological cure, supporting current empiric treatment.

## 1. Introduction

Pelvic inflammatory disease (PID) involves inflammation of the upper female genital tract, predominantly affecting sexually active women of reproductive age. It is associated with substantial morbidity, including tubal infertility and ectopic pregnancy [1]. Tubo‐ovarian abscess (TOA), a severe complication of PID, is an inflammatory adnexal mass, with substantial risk of rupture and potentially fatal consequences [2]. PID and TOA result from ascending microorganisms from the vagina or endocervix. However, microbiological assessment of PID and TOA is most commonly performed using samples from the lower genital tract, as direct sampling of the upper genital tract is invasive and rarely performed. Although lower genital tract organisms may contribute to ascending infection, detection in vaginal or cervical specimens does not necessarily reflect the microbial composition of the upper genital tract [3]. *Chlamydia trachomatis* and *Neisseria gonorrhoeae* are frequently implicated as causative pathogens; however, the etiology is unidentified in up to 70% of the PID cases [4–6]. The role of other microorganisms, such as *Mycoplasma genitalium*, remains incompletely understood. A meta‐analysis reported a modest association between *M. genitalium* and PID (pooled OR 1.67, 95% CI 1.24–2.24) [7]; though several included studies found no significant link [8–11], highlighting the heterogenous evidence. International diagnostic guidelines reflect this inconsistency: Testing for *M. genitalium* in suspected PID cases is recommended in Europe [12], the United Kingdom [13], and Australia [14] but not in the Netherlands [15] or the United States [16].

The therapeutic relevance of *M. genitalium* in PID and TOA also remains uncertain. Dutch hospital guidelines advise ofloxacin or levofloxacin with metronidazole if *N. gonorrhoeae* is excluded [15], yet both fluoroquinolones show only moderate *in vitro* activity against *M. genitalium* [17–19]. At the Centre for Sexual Health (CSH), doxycycline plus metronidazole is prescribed. Although recent data suggest that this combination may offer modest efficacy against *M. genitalium* [20], eradication often requires antibiotics with greater intrinsic activity. The 2021 European guideline for the management of *M. genitalium* recommends as the first‐line antibiotic within a resistance‐guided treatment strategy for uncomplicated infection [21]. However, resistance to azithromycin has been increasing, with many European countries now reporting rates above 50% [22]. For azithromycin‐resistant *M. genitalium* infections, moxifloxacin is the advised therapy [21]. Alarmingly, resistance to moxifloxacin has also emerged, with rates up to 34% in Europe [22]. Moreover, fluoroquinolones are considered last‐resort antibiotics and have been associated, although rarely, with serious and potentially irreversible adverse effects [23].

These discrepancies and increasing antibiotic resistance underscore the need to clarify the role of *M. genitalium* in PID and TOA and to evaluate the effectiveness of current treatment regimens. Accordingly, the objectives were twofold: (1) to evaluate the involvement of *M. genitalium, C. trachomatis*, and *N. gonorrhoeae* in PID and TOA among patients in a high‐income European setting and (2) to assess the clinical and microbiological effectiveness of current standard treatment regimens used in the Netherlands for women diagnosed with PID or TOA, irrespective of STI status.

## 2. Materials and Methods

### 2.1. Participant Enrollment

In this multicenter prospective cohort study, participants were enrolled between 2021 and 2023 at four healthcare institutions located in different cities in the Netherlands: OLVG Hospital in Amsterdam, Meander Medical Centre in Amersfoort, Flevo Hospital in Almere, and the CSH in Amsterdam. Women with a clinical diagnosis of PID and/or TOA were invited to participate in the study. PID diagnosis was established according to the criteria outlined in the Federation Medical Specialists (FMS) clinical protocol [15], which requires lower abdominal pain accompanied by at least one of the following symptoms: abnormal vaginal discharge, fever, deep dyspareunia, pelvic tenderness, or intermenstrual bleeding. In cases of suspected TOA, diagnosis was supported by a transvaginal ultrasound. Eligible participants were ≥ 18 years and able to comprehend the study information in Dutch or English. Exclusion criteria comprised current pregnancy, recent cervical procedures (past month), prior documented *M. genitalium* infection, or allergy to the antibiotics prescribed in the study. All participants provided written informed consent prior to enrollment.

### 2.2. Study Procedures

During the baseline visit (Day 0), clinicians conducted a physical and pelvic examination, recorded baseline clinical presentation, and routinely collected vaginal swabs for the detection of *C. trachomatis* and *N. gonorrhoeae,* in accordance with standard diagnostic guidelines [15]. Testing for *M. genitalium* was additionally performed for research purposes only. At Week 2, participants were requested to self‐collect and postvaginal swabs for *M. genitalium* testing. A follow‐up visit at Week 4 postbaseline included a repeat physical and pelvic examination and collection of a vaginal swab for *M. genitalium* testing; participants with *C. trachomatis* or *N. gonorrhoeae* at baseline were retested at Weeks 2 and 4. Persistent *M. genitalium* at Week 4 was reported to physicians if treatment was indicated. Blood samples were collected at both baseline and the Week‐4 follow‐up visit to measure C‐reactive protein (CRP) as a marker for inflammation. Participants were asked to complete self‐reported symptom diaries on Days 1, 3, 5, 7, 14, and 28, rating most symptoms on a 0–5 scale (0 = *none/normal*, 5 = *a lot/very [different]*), with fever and vomiting recorded using a binary yes/no scale. The diaries were completed on paper, with a template provided in Supporting Figure 1.

### 2.3. Treatment Regimes

All participants were intended to be managed in accordance with the current Dutch clinical guidelines for hospitals and the CSH. Participants enrolled at hospitals were advised to be treated with 400 mg 2 dd oral ofloxacin or 500 mg 1 dd intravenous levofloxacin plus 500 mg 2 dd oral metronidazole, all for a duration of 14 days, in line with the FMS clinical protocol [15]. Participants enrolled at the CSH were recommended to be treated with doxycycline 2 dd 100 mg and metronidazole 2 dd 500 mg for a duration of 14 days, according to local guidelines. At all study sites, a single intramuscular dose of 500 or 1000 mg ceftriaxone was additionally recommended in cases of suspected or confirmed *N. gonorrhoeae* infection.

### 2.4. Microbiological Testing

Vaginal swabs collected at baseline, and at Weeks 2 and 4, were analyzed for *M. genitalium*, *C. trachomatis*, and *N. gonorrhoeae* in accordance with local diagnostic protocols. Samples collected at OLVG, Flevo Hospital, and CSH were tested using transcription‐mediated amplification (TMA) assays (Hologic, San Diego, California, USA). Positive *M. genitalium* specimens underwent confirmatory testing using the MgPa qPCR assay [24]. At the Meander Medical Center, samples were tested for *C. trachomatis* and *N. gonorrhoeae* using the m2000 CT/NG assay (Abbott Molecular, Abbott Park, Illinois, USA) until August 31, 2022, after which the Hologic TMA assay was used for the remainder of the study period. The presence of *M. genitalium* in these samples was assessed using the Mg‐Mares qPCR assay [25].

### 2.5. Study Outcomes

The primary outcome of the study was clinical cure by treatment of PID and TOA at Week 4, defined as the absence of fever (oral temperature of < 38°C) and vomiting, a CRP below 10.0 mg/L, and at least a 70% reduction in the combined clinical symptom score. Participants who did not meet all three criteria were classified as not having achieved clinical cure in the study. In case one of these variables was missing, clinical cure was undetermined. Clinical symptoms included abdominal pain, shivering, dysuria, abnormal vaginal discharge, intermenstrual bleeding, malaise, nausea, and fatigue and were self‐reported by participants using a symptom diary. The combined clinical symptom score (ranging 0–40) was derived by summing the individual scores of these eight symptoms. If any individual symptom score was missing for a given day, the combined clinical symptom score was not calculated for that day. The secondary outcome was microbiological cure after treatment which was defined as a negative test result for *M. genitalium, C. trachomatis*, and *N. gonorrhoeae* at week 4.

### 2.6. Statistical Analysis

Participants were excluded from the baseline analysis if they had a confirmed diagnosis other than PID and/or TOA or lacked an *M. genitalium* test result on the day of enrollment. Differences in microbiological and clinical outcomes, both over time and between participant groups, were assessed using the chi‐square test, Fisher’s exact test, Mann–Whitney *U* test, Wilcoxon signed‐rank test, and McNemar’s test. Statistical analyses were conducted using Stata (Version 17.0, StataCorp, College Station, Texas, USA).

### 2.7. Details of Ethical Approval

The study was approved by the Amsterdam Medical Centre Ethics Board (NL72299.018.19) and was monitored by an independent monitor. Written consent was obtained from participants after being informed verbally about the study. The study was conducted in accordance with the Medical Research Involving Human Subjects Act (WMO) of Dutch law.

## 3. Results

### 3.1. Demographics and Clinical Presentation of Participants During Baseline Visit

**FIGURE 1:**
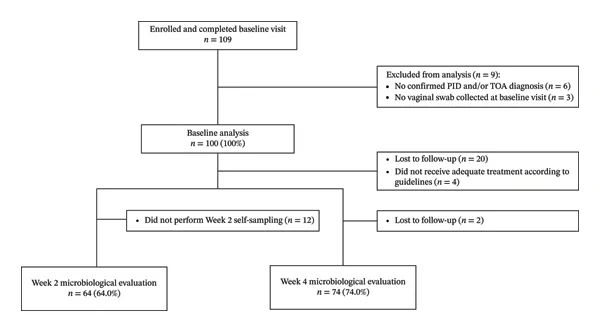
Flowchart participant enrollment and follow‐up throughout the study. PID, pelvic inflammatory disease; TOA, tubo‐ovarian abscess.

From March 2021 through December 2023, 109 women with suspected PID and/or TOA were enrolled in the study (Figure 1). Nine participants were excluded from further analysis due to the absence of a confirmed PID and/or TOA diagnosis (*n* = 6) or lack of a vaginal swab collected at the baseline visit (*n* = 3). As a result, 100 participants were included in the final analysis. Of these, 64 (64.0%) self‐collected a vaginal swab at Week 2, and 74 (74.0%) returned for the follow‐up visit in Week 4.

Main participant characteristics are summarized in Table 1. The median age was 30 years (interquartile range [IQR], 25–38), and most participants (97.0%) were included at hospitals, with only 3.0% enrolled at the CSH. Nearly half (48.0%) reported the Netherlands as their region of origin, and 54.0% had attained a college or university level of education. Human immunodeficiency virus (HIV) status was available for 25.0% of participants, of whom none were living with HIV. The median number of sexual partners in the last 3 months was 1 (IQR, 1‐1). A prior history of PID or TOA was documented in 4.0% of participants.

**TABLE 1:** Participant characteristics at baseline.

	Participants (*n* = 100)
Age (years), median (IQR)	30 (25–38)
Centre of inclusion	
Centre for Sexual Health	3 (3.0)
Hospital	97 (97.0)
Region of origin	
The Netherlands	48 (48.0)
Europe, outside the Netherlands	8 (8.0)
Africa	3 (3.0)
Asia	8 (8.0)
North and Central America	1 (1.0)
South America	4 (4.0)
Unknown	28 (28.0)
Educational level	
Primary	1 (1.0)
Secondary	18 (18.0)
College/university	54 (54.0)
Unknown	27 (27.0)
Living with HIV	
No	25 (25.0)
Yes	0 (0)
Unknown	75 (75.0)
Received money or goods in exchange for sex	
No	76 (76.0)
Yes	1 (1.0)
Unknown	23 (23.0)
No. of sexual partners in previous 3 months, median (IQR)	1 (1‐1)^a^
History of PID and/or TOA	
No	93 (93.0)
Yes	4 (4.0)
Unknown	3 (3.0)
Sexually transmitted infection prevalence	
*M. genitalium*	4 (4.0)
*C. trachomatis*	6 (6.0)
*N. gonorrhoeae*	9 (9.0)
*M. genitalium* and *C. trachomatis*	1 (1.0)
*C. trachomatis* and *N. gonorrhoeae*	1 (1.0)
*M. genitalium* and *N. gonorrhoeae*	2 (2.0)
None	77 (77.0)
Confirmed diagnosis	
PID	78 (78.0)
TOA + PID	22 (22.0)
Antibiotic regimen given	
Ofloxacin‐ or levofloxacin‐based antibiotic regimen^b^	88 (88.0)
Doxycyclin‐based antibiotic regimen^c^	8 (8.0)
Other^d^	4 (4.0)

*Note:* Data are presented as number (%) unless otherwise indicated.

Abbreviations: HIV, human immunodeficiency virus; PID, pelvic inflammatory disease; TOA, tubo‐ovarian abscess.

^a^Data missing for 16 participants.

^b^Ofloxacin/levofloxacin antibiotic regimen comprised 400 mg 2 dd oral ofloxacin or 500 mg 1 dd intravenous levofloxacin in combination with 500 mg 2 dd oral metronidazole, all for 14 days.

^c^Doxycyclin‐based antibiotic regimen comprised doxycycline 2 dd 100 mg and metronidazole 2 dd 500 mg, both for 14 days. For both the fluoroquinolone and doxycycline regimens, a single intramuscular dose of ceftriaxone, 1000 mg or 500 mg, respectively, could additionally be prescribed in cases of suspected or confirmed *N. gonorrhoeae* infection.

^d^Other included the use of ciprofloxacin or amoxicillin instead of the recommended fluoroquinolones (ofloxacin or levofloxacin), or a oxifloxacin/levofloxacin antibiotic regimen administered for fewer than 14 days. IQR, interquartile range.

The prevalence of monoinfections with *M. genitalium, C. trachomatis*, and *N. gonorrhoeae* at baseline was 4.0%, 6.0%, and 9.0%, respectively (Table 1). Coinfections were observed in four participants: one case each of *M. genitalium* with *C. trachomatis* and *C. trachomatis* with *N. gonorrhoeae,* and two cases of *M. genitalium* with *N. gonorrhoeae*. The vast majority of participants (77.0%) tested negative for all three pathogens. Among all participants, 78 (78.0%) were diagnosed with PID and 22 (22.0%) with both PID and TOA. The majority (88.0%) received an ofloxacin‐ or levofloxacin‐based antibiotic regimen, exclusively prescribed at hospitals. Eight (8.0%) participants received a doxycycline‐based regimen, including three from the CSH and five from hospitals. Four participants received treatment regimens that deviated from the FMS clinical protocol for hospitals [15] or local CSH guidelines and were, therefore, excluded from follow‐up analyses at Weeks 2 and 4.

**FIGURE 2:**
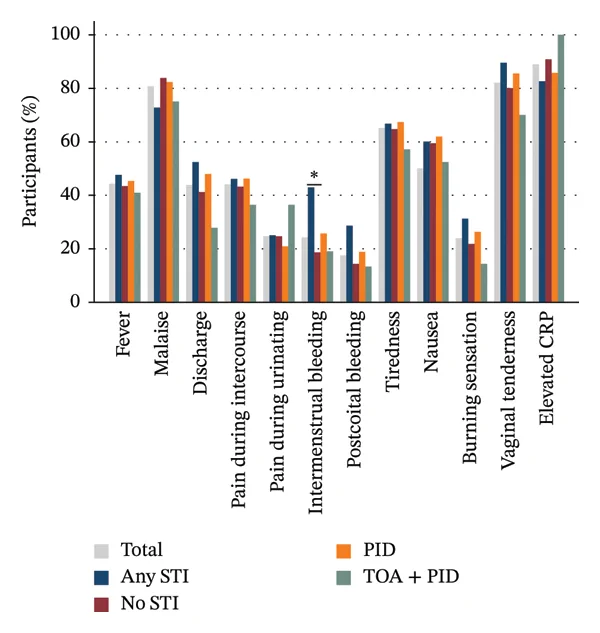
Baseline clinical presentation of participants during the baseline visit (Day 0), stratified by STI status and diagnosis. The “Total” group includes the total study population (*n* = 100); the “Any STI” group includes participants who tested positive for at least one STI, including the coinfections (*n* = 23); the “No STI” group includes participants who tested negative for all STIs (*n* = 77); the “PID” group includes participants who were diagnosed with PID (*n* = 78); and the “TOA + PID” group includes participants who were diagnosed with both TOA and PID (*n* = 22). Bars represent the percentage of participants reporting each symptom or sign, calculated from the number of participants with available (nonmissing) data. Either the chi‐square test or Fisher’s exact test was performed to indicate statistically significant differences in the frequency of experienced symptoms and signs between STI status groups (any STI or no STI) and diagnosis groups (PID or TOA + PID) (^∗^, *p* < 0.05). STI, sexually transmitted infection; PID, pelvic inflammatory disease; TOA, tubal‐ovarian abscess.

The baseline clinical presentation (Day 0) of participants, stratified by STI status (any STI or no STI) and diagnosis (PID or TOA + PID), is shown in Figure 2 and Table S1. Overall, the most commonly reported symptoms and signs were elevated CRP (> 10 mg/L) (88.9%), vaginal tenderness (82.0%), malaise (80.7%), and tiredness (65.1%). Intermenstrual bleeding was reported significantly more frequently among participants positive for any STI (42.9%) compared to those without an STI (18.6%) (*p* = 0.023). Among participants with an STI, infection with *N. gonorrhoeae* was associated with a significantly higher proportion of elevated CRP (100%) compared with *M. genitalium* or *C. trachomatis* infections (63.6%) (*p* = 0.037), with no other symptoms and signs differing significantly between these groups. There were no statistically significant differences in presenting signs and symptoms between participants diagnosed with PID and those with both TOA and PID.

### 3.2. Microbiological Outcomes Overtime

Participants who tested positive for any pathogen at baseline were subjected to retesting in Weeks 2 and 4 (Table 2). No *M. genitalium* infections were detected at either follow‐up visit, suggesting a 100% clearance. For *C. trachomatis*, 40% of the retested individuals remained positive at Week 2, with no cases detected by Week 4. In the case of *N. gonorrhoeae,* 40.0% of participants were still positive at Week 2, decreasing to 9.1% by Week 4. The decline in *N. gonorrhoeae* prevalence over time was statistically significant (*p* = 0.004), whereas changes in *M. genitalium* and *C. trachomatis* prevalence were not, although interpretation is limited by the small number of total cases and missing follow‐up data.

**TABLE 2:** Microbiological testing overtime.

	Baseline	Week 2	Week 4	*p* value^d^
*M. genitalium*	7/100 (7.0)^a^	0/3 (0)	0/4 (0)	0.125
*C. trachomatis*	8/100 (8.0)^b^	2/5 (40.0)	0/5 (0)	0.063
*N. gonorrhoeae*	12/100 (12.0)^c^	4/10 (40.0)	1/11 (9.1)	0.004

*Note:* Data are presented as the number of positive cases out of the total number of participants tested (%). Denominators reflect the number positives at baseline who were retested for each pathogen.

^a^One *M. genitalium*/*C. trachomatis* coinfection and two *M. genitalium*/*N. gonorrhoeae* coinfections.

^b^One *M. genitalium*/*C. trachomatis* coinfection and one *C. trachomatis*/*N. gonorrhoeae* coinfection.

^c^One *C. trachomatis*/*N. gonorrhoeae* coinfection and two *M. genitalium*/*N. gonorrhoeae* coinfections. Only participants who tested positive for a given pathogen at baseline are included in the results of Weeks 2 and 4.

^d^Exact McNemar’s test was performed to indicate significant differences in the proportion of positive cases between baseline and Week 4.

### 3.3. Clinical Outcomes Overtime

**FIGURE 3:**
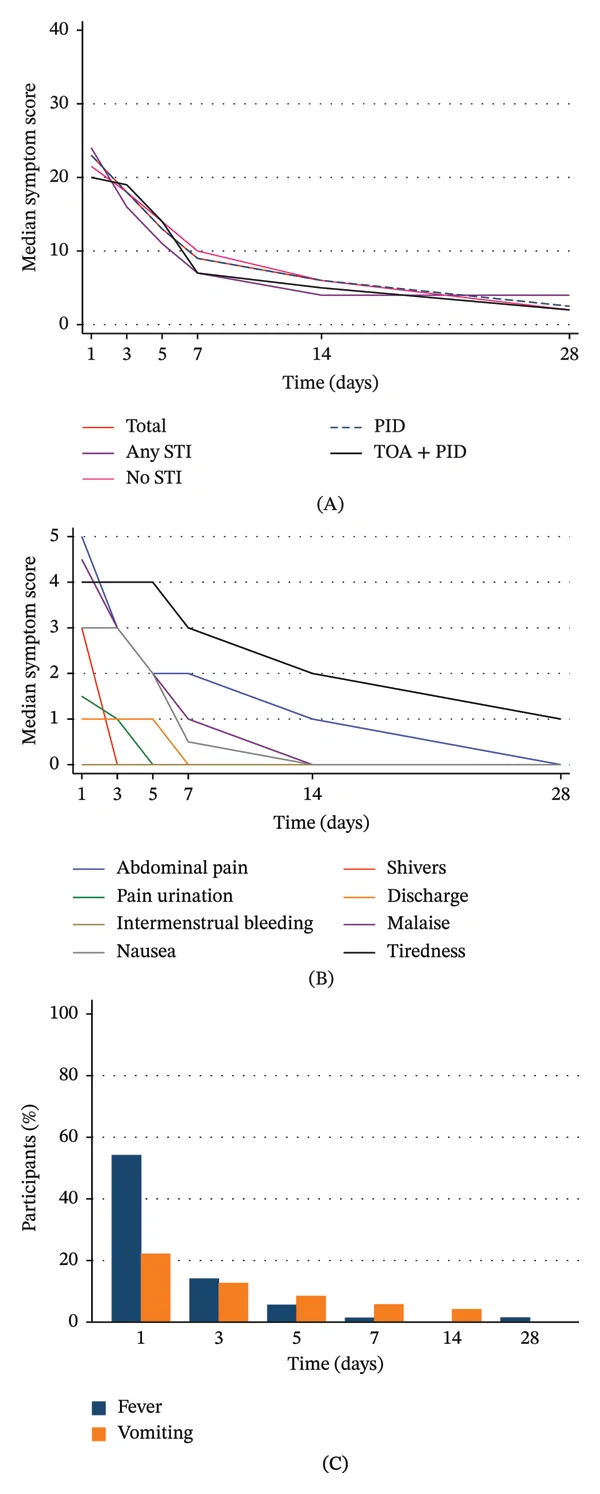
Trends in self‐reported symptom severity overtime. (A) Combined symptom score representing the sum of severity ratings across eight assessed symptoms as was documented in the self‐reported dairy, stratified by STI status and diagnosis. (B) Median severity scores over time for individual symptoms, rated using a 0–5 Likert scale (0 = *no symptom*, 5 = *most severe*). (C) Proportion of participants (%) reporting fever or vomiting. STI, sexually transmitted infection; PID, pelvic inflammatory disease; TOA, tubal‐ovarian abscess.

The combined symptom score, calculated as the sum of the eight individual symptom scores, decreased by 91.3%, from a median of 23 (IQR, 16–27) on Day 1‐2 (IQR, 0.5‐6) on Day 28 (Figure 3A and Table S2). No statistically significant differences in the reduction of combined symptom scores were observed between groups defined by STI status (*p* = 0.906) or diagnosis (*p* = 0.702).

Self‐reported symptom severity demonstrated significant improvement across all symptoms, except intermenstrual bleeding (could not be determined), over the course of follow‐up (Figure 3B and Table S3). Abdominal pain, shivers, pain during urination, unusual discharge, intermenstrual bleeding, malaise, and nausea all showed a complete reduction to a median score of 0 by Day 28. Among these, abdominal pain had the highest median baseline score of 5 (IQR 4‐5). Tiredness was the only symptom that persisted on Day 28, with a median score of 1 (IQR 0–2), although it showed a statistically significant 75% reduction from baseline (*p* < 0.001). Fever and vomiting resolved in nearly all participants by Day 28, except for one participant (1.5%) in whom fever persisted (Figure 3C).

At baseline (Day 0), the overall median CRP level was 82 mg/L (IQR, 39–149). Participants diagnosed with both TOA and PID had significantly higher CRP levels compared to those with PID alone (*p* < 0.001) (Table 3). In contrast, no significant differences in baseline CRP levels were observed for STI status (*p* = 0.865). After 4 weeks of follow‐up, CRP levels significantly decreased across all participants, with a median reduction of 84.0 mg/L to a final median value of 1.6 mg/L (*p* < 0.001). Subgroup analyses confirmed similarly significant reductions in CRP levels among participants positive for an STI (median decrease: 77.5 mg/L, *p* < 0.001), those negative for an STI (84 mg/L, *p* < 0.001), those diagnosed with PID (66.2 mg/L, *p* < 0.001), and those with both TOA and PID (178 mg/L, *p* < 0.001).

**TABLE 3:** C‐reactive protein levels overtime.

	All participants (*n* = 99)^a^	Any STI (*n* = 23)	No STI (*n* = 76)	*p* value STI status^h^	PID (*n* = 77)	TOA + PID (*n* = 22)	*p* value diagnosis^g^
CRP at baseline, median (IQR)	82 (39–149)	72 (23–184)	84.5 (41.5–148)	0.865	67 (28–142)	148 (92–248)	< 0.001
CRP at Week 4, median (IQR)	1.6 (0.6–3.7)^f^	1.7 (0.7–3.4)^c^	1.6 (0.6–3.7)^d^	0.770	1.1 (0.6–3.0)^e^	2 (0.8–6.2)^b^	0.271
CRP difference from baseline	−84.0	−77.5	−84	0.801	−66.2	−178	< 0.001
*p* value^h^	< 0.001	< 0.001	< 0.001	—	< 0.001	< 0.001	—

*Note:* IQR, interquartile range.

Abbreviations: CRP, C‐reactive protein; PID, pelvic inflammatory disease; STI, sexually transmitted infection; TOA, tubal‐ovarian abscess.

^a^One case missing.

^b^Seven cases missing.

^c^Nine cases missing.

^d^Twenty‐two cases missing.

^e^Twenty‐four cases missing.

^f^Thirty‐one cases missing.

^g^Mann–Whitney *U* test was performed to indicate statistically significant differences in median CRP levels for STI status (any STI or no STI) and diagnosis (PID or TOA + PID).

^h^Wilcoxon signed‐rank test was performed to indicate significant differences in median CRP levels between baseline and Week 4.

Although clinical cure was assessed in all included participants, it could only be conclusively determined in 52 participants with complete follow‐up data. Overall, 35/52 participants (67.3%) achieved clinical cure over the study period of 4 weeks (Table 4). Among the 17 participants who did not achieve clinical cure, 1 (5.9%) exhibited persistent fever, 2 (11.8%) did not meet the CRP threshold of < 10 mg/L, and 14 (82.4%) had less than a 70% reduction in their total clinical symptom score. Clinical symptom scores on Days 1 and 28 for participants with less than a 70% reduction are presented in Table S4. Among participants without an STI at baseline, 28 (68.3%) demonstrated clinical cure, compared to 7 (63.6%) of those with an STI. Regarding diagnosis, clinical cure was observed in 67.5% of participants with both TOA and PID, compared to 67.5% among those with PID. However, there was no statistically significant association between clinical cure and either STI status (*p* = 1.000) or diagnosis (*p* = 1.000).

**TABLE 4:** Clinical cure stratified by STI status and diagnosis.

	Clinical cure determined n	Clinical cure achieved *n* (%)	*p* value^a^
Total	52	35 (67.3)	—

Any STI	11	7 (63.6)	1.000
No STI	41	28 (68.3)	

PID	40	27 (67.5)	1.000
TOA + PID	12	8 (66.7)	

*Note:* Clinical cure was defined as the absence of fever and vomiting, a CRP below 10.0 mg/L, and at least a 70% reduction in the combined clinical symptom score. “Clinical cure determined” refers to participants for whom a clinical outcome assessment was completed. “Clinical cure achieved” indicates participants who showed evidence of cure based on predefined definition.

Abbreviations: PID, pelvic inflammatory disease; STI, sexually transmitted infection; TOA, tubal‐ovarian abscess.

^a^Fisher’s exact test was performed to indicate significant differences in the proportion of participants with clinical cure by STI status (any STI or no STI) and diagnosis (PID or TOA + PID).

## 4. Discussion

### 4.1. Main Findings

In this multicenter prospective cohort study, we investigated the involvement of *M. genitalium*, *C. trachomatis*, and *N. gonorrhoeae* in women diagnosed with PID and/or TOA and assessed the effectiveness of standard treatment regimens in achieving clinical and microbiological cure. Our findings indicate that the majority of women diagnosed with PID and/or TOA tested negative for the three pathogens, highlighting the multifactorial etiology of PID. Despite this, we observed that standard treatment regimens were associated with substantial clinical cure and microbiological clearance, demonstrating overall effectiveness of treatment across etiological spectra.

### 4.2. Strength and Limitations

This multicenter prospective cohort study provides a broad and clinically relevant perspective on the management of PID and TOA across diverse healthcare settings and patient populations, supporting the generalizability. However, the relatively short follow‐up period of 4 weeks limited the ability to evaluate long‐term outcomes, including sustained microbiological and clinical cure, as well as potential effects on fertility. A key strength is the inclusion of *M. genitalium* testing in addition to *C. trachomatis* and *N. gonorrhoeae*, allowing for a more comprehensive exploration of the microbial etiology of PID and TOA. Nonetheless, the low prevalence of these pathogens, combined with the limited sample size, reduced the statistical power to detect pathogen‐specific treatment effects. Furthermore, *Trichomonas vaginalis* and bacterial vaginosis (BV) were not assessed in the present analysis. Given the relatively low prevalence of *T. vaginalis* in the Netherlands [26], its impact on the overall findings is expected to be limited. BV will be examined in a planned secondary analysis, enabling a more comprehensive assessment of its potential association with PID and related clinical outcomes. The study’s concurrent assessment of microbiological and clinical endpoints enhances the interpretability of treatment responses for PID and TOA, although reliance on a clinical case definition for PID, which lacks optimal specificity and sensitivity, introduced a risk of diagnostic misclassification. Lastly, while the real‐world design strengthens the applicability of the findings to routine practice, the substantial loss to follow‐up, particularly among participants with *M. genitalium* infection, limits the robustness of the observed 100% clearance rate and warrants cautious interpretation.

### 4.3. Interpretation

The prevalence of *M. genitalium* in our cohort, including coinfections, was 7.0%, which is lower than the 13%–16% reported in several studies of women with suspected PID [6, 27–29]. In the PEACH study, *M. genitalium* was identified in 15.0% of participants using both cervical and endometrial swabs [30]. Similarly, *C. trachomatis* prevalence in our cohort (8.0%) was below the 10%–38% range reported in other studies [28, 31–33]. Although a wide range of *N. gonorrhoeae* prevalence has been reported, the rate observed in our cohort (12.0%) was relatively low, positioned at the lower end of the previously reported spectrum (2%–80%) [28, 31–33]. These lower detection rates may partly reflect the age distribution of our cohort (median 30 years; only 25% were under 25, the group at highest STI risk). Nevertheless, our data align with another study reporting that up to 70% of PID cases lack an etiology related to the traditional pathogens *C. trachomatis* and *N. gonorrhoeae* [28]. In our cohort, 77% of participants tested negative for *M. genitalium, C. trachomatis*, and *N. gonorrhoeae*, further supporting the polymicrobial and multifactorial nature of PID and TOA pathogenesis. Other implicated organisms include BV‐associated organisms (e.g., *Gardnerella vaginalis* and *Sneathia* species) and enteric or respiratory organisms (e.g., *Escherichia coli* and *Staphylococcus aureus*) [33–36]. Whether these act as causal agents or opportunists in a disrupted upper genital tract environment remains uncertain [31]. Nonetheless, our findings underscore the need for broader diagnostic approaches in PID beyond conventional STI screening.

Encouragingly, we observed a 100% clearance rate of *M. genitalium* and *C. trachomatis,* and near‐complete clearance of *N. gonorrhoeae* by Week 4 posttreatment. Residual detection of *N. gonorrhoeae* may reflect the presence of nonviable DNA or reinfection rather than treatment failure [37]. However, the high clearance rates should be interpreted with caution due to the limited number of STI‐positive cases and substantial loss to follow‐up, particularly among *M. genitalium*‐positive patients, limiting the ability to draw definitive conclusions regarding microbial eradication.

The low prevalence of *M. genitalium* in our study does not support a major causative role in PID. Standard empiric treatment regimens are effective against *C. trachomatis, N. gonorrhoeae*, and anaerobes [36] but provide suboptimal coverage for *M. genitalium*. Although *M. genitalium* demonstrates higher susceptibility to moxifloxacin [38], our findings have demonstrated that routine incorporation of moxifloxacin into empiric PID treatment strategies is not warranted in all settings. Given rising macrolide and fluoroquinolone resistance and the possible role of *M. genitalium* in upper genital tract infections, targeted testing and resistance‐guided treatment, particularly for persistent or recurrent cases, may better support antimicrobial stewardship.

Clinically, we observed significant improvement in symptoms and signs across most participants. The median combined clinical symptom score declined by over 90% during the four‐week follow‐up period, with near‐complete resolution of most symptoms. Parallel to symptom improvement, CRP fell from a median of 82 mg/L at baseline to 1.6 mg/L by Week 4. Although baseline CRP was higher in participants with TOA and PID than in those with PID alone, both groups showed similar resolution patterns. Two‐thirds of participants with complete follow‐up met the predefined criteria for clinical cure, consistent across STI‐positive and ‐negative cases. This proportion appears lower than the 93% cure rate reported in another study [20], likely because our definition included not only abdominal tenderness but also nonspecific symptoms such as fatigue, which may persist beyond 30 days or be unrelated to PID. Overall, our findings suggest that empiric treatment regimens remain broadly effective regardless of the etiological agent.

## 5. Conclusion

The majority of cases lacked an identifiable STI etiology in lower genital tract samples, reinforcing the polymicrobial and multifactorial nature of PID and TOA. However, clinical and microbiological outcomes were generally favorable, with high pathogen clearance rates and substantial symptom improvement. These findings support the continued use of current empiric therapies while not excluding the potential value of targeted testing and resistance‐guided treatment for STIs in selected cases, in line with antimicrobial stewardship principles.

## Author Contributions

Project conception: Joyce F. Braam and Alje P. van Dam. Conceptualization and methodology: Joyce F. Braam, Alje P. van Dam, Sylvia M. Bruisten, Clarissa E. Vergunst, Roel H. T. Nijhuis, Arien Bukman, Paul J. M. van Kestere, Paul Gruteke, and Merel van Doorenmaalen. Study coordination: Nikki Adriaens and Joyce F. Braam. Data analysis: Nikki Adriaens. Supervision: Vita W. Jongen and Alje P. van Dam. Manuscript drafting: Nikki Adriaens. Editing and revision: Joyce F. Braam, Alje P. van Dam, Sylvia M. Bruisten, Clarissa E. Vergunst, Vita W. Jongen, Roel H. T. Nijhuis, Arien Bukman, Paul J. M. van Kesteren, Paul Gruteke, and Merel van Doorenmaalen.

## Funding

This study was supported by an internal research grant provided by the OLVG hospital.

## Disclosure

Nikki Adriaens affirms that this manuscript is an honest, accurate, and transparent account of the study being reported, that no important aspects of the study have been omitted, and that any discrepancies from the study as planned have been explained. All authors have read and approved the final version of the manuscript. Nikki Adriaens had full access to all of the data in this study and takes complete responsibility for the integrity of the data and the accuracy of the data analysis.

## Conflicts of Interest

The authors declare no conflicts of interest.

## Supporting Information

Additional supporting information can be found online in the Supporting Information section.

## Supporting information


**Supporting Information** File S1: Example of symptom diary. Table S1: Baseline clinical presentation (Day 0) of participants stratified by STI status and diagnosis. Table S2: Combined symptom score stratified by STI status and diagnosis. Table S3: Clinical outcomes based on self‐reported symptom diary. Table S4: Clinical symptom scores of participants with < 70% reduction in symptom scores.

## Data Availability

The data that support the findings of this study are available on request from the corresponding author. The data are not publicly available due to privacy or ethical restrictions.
