# Supplementary material for: Limited Role of Mycoplasma genitalium, Chlamydia trachomatis, and Neisseria gonorrhoeae in Pelvic Inflammatory Disease and Tubo‐Ovarian Abscess in the Netherlands: A Multicenter Prospective Cohort Study
Source: Obstet Gynecol Int. 2026 Sep 12;2026:3810643. doi: 10.1155/ogi/3810643 (PMC13570452; doi:10.1155/ogi/3810643)
Supplement: Supplementary file 1 [file OGI-2026-3810643-s001.docx]

**Supplementary Materials**

**Supplementary Figure 1. Example of symptom diary**

**I have had the following complaints / problems in the last 24 hours:**

Pain in lower abdomen	none	0	1	2	3	4	5	a lot

Cold shivers	none	0	1	2	3	4	5	a lot

Pain when urinating	none	0	1	2	3	4	5	a lot

Vaginal discharge	normal	0	1	2	3	4	5	very different

Vaginal blood loss	none	0	1	2	3	4	5	a lot

Pain during intercourse	none	0	1	2	3	4	5	a lot	n.a.

Blood loss during intercourse	none	0	1	2	3	4	5	a lot	n.a.

I have had a fever in the last 24 hours:	Yes	No
Degrees:	……

I vomited in the last 24 hours:	Yes	No

Number of times:	……

**In the past 24 hours I have felt:**

Sick	not	0	1	2	3	4	5	very

Nauseous	not	0	1	2	3	4	5	very

Tired	not	0	1	2	3	4	5	very

Below you can write down other complaints / problems and indicate how bad it was with a number:

………………………	not	0	1	2	3	4	5	very

………………………	not	0	1	2	3	4	5	very

I feel better than yesterday	no	0	1	2	3	4	5	a lot

**Table S1.** Baseline clinical presentation (day 0) of participants stratified by STI status and diagnosis.

|  | **Total**  **(n=100)** | **Any STI**  **(n=23)** | **No STI**  **(n=77)** | ***P*-value**  **STI status** | **PID**  **(n=78)** | **TOA and PID (n=22)** | ***P*-value diagnosis** |
| --- | --- | --- | --- | --- | --- | --- | --- |
| **Fever**  No  Yes  Unknown | 54 (55.7)  43 (44.3)  3 | 11 (52.4)  10 (47.6)  2 | 43 (56.6)  33 (43.4)  1 | 0.732^a^ | 41 (54.7)  34 (45.3)  3 | 13 (59.1)  9 (40.9)  0 | 0.713^a^ |
| **Malaise**  No  Yes  Unknown | 17 (19.3)  71 (80.7)  12 | 6 (27.3)  16 (72.7)  1 | 11 (16.7)  55 (83.3)  11 | 0.275^a^ | 12 (17.7)  56 (82.3)  10 | 5 (25.0)  15 (75.0)  2 | 0.464^a^ |
| **Unusual discharge**  No  Yes  Unknown | 50 (56.2)  39 (43.8)  11 | 10 (47.6)  11 (52.4)  2 | 40 (58.8)  28 (41.2)  9 | 0.366^a^ | 37 (52.1)  34 (47.9)  7 | 13 (72.2)  5 (27.8)  4 | 0.125^a^ |
| **Pain during intercourse**  No  Yes  Unknown | 28 (56.0)  22 (44.0)  50 | 7 (53.9)  6 (46.1)  10 | 21 (56.8)  16 (43.2)  40 | 0.856^a^ | 21 (53.9)  18 (46.2)  39 | 7 (63.6)  4 (36.4)  11 | 0.734^b^ |
| **Pain during urination**  No  Yes  Unknown | 67 (75.3)  22 (24.7)  11 | 15 (75 .0)  5 (25.0)  3 | 52 (75.4)  17 (24.6)  8 | 0.974^a^ | 53 (79.1)  14 (20.9)  11 | 14 (63.6)  8 (36.4)  0 | 0.144^a^ |
| **Intermenstrual bleeding**  No  Yes  Unknown | 69 (75.8)  22 (24.2)  9 | 12 (57.1)  9 (42.9)  2 | 57 (81.4)  13 (18.6)  7 | 0.023^a^ | 52 (74.3)  18 (25.7)  8 | 17 (81.0)  4 (19.0)  1 | 0.772^b^ |
| **Postcoital bleeding**  No  Yes  Unknown | 52 (82.5)  11 (17.5)  37 | 10 (71.4)  4 (28.6)  9 | 42 (85.7)  7 (14.3)  28 | 0.447^b^ | 39 (81.3)  9 (18.8)  30 | 13 (86.7)  2 (13.3)  7 | 1.000^b^ |
| **Tiredness**  No  Yes  Unknown | 23 (34.9)  43 (65.2)  34 | 5 (33.3)  10 (66.7)  8 | 18 (35.3)  33 (64.7)  26 | 0.889^a^ | 17 (32.7)  35 (67.3)  26 | 6 (42.9)  8 (57.1)  8 | 0.479^a^ |
| **Nausea**  No  Yes  Unknown | 34 (34.0)  50 (50.0)  16 | 8 (40.0)  12 (60.0)  3 | 26 (40.6)  38 (59.4)  13 | 0.801^a^ | 24 (38.1)  39 (61.9)  15 | 10 (47.6)  11 (52.4)  1 | 0.293^a^ |
| **Burning sensation**  No  Yes  Unknown | 54 (76.1)  17 (23.9)  29 | 11 (68.8)  5 (31.2)  7 | 43 (78.2)  12 (21.8)  22 | 0.437^a^ | 42 (73.7)  15 (26.3)  21 | 12 (85.7)  2 (14.3)  8 | 0.493^b^ |
| **Vaginal tenderness**  No  Yes  Unknown | 16 (18.0)  73 (82.0)  11 | 2 (10.5)  17 (89.5)  4 | 14 (20.0)  56 (80.0)  7 | 0.506^b^ | 10 (14.5)  59 (85.5)  9 | 6 (30.0)  14 (70.0)  2 | 0.112^a^ |
| **Elevated CRP (>10 mg/L)**  No  Yes  Unknown | 11 (11.1)  88 (88.9)  1 | 4 (17.4)  19 (82.6)  0 | 7 (9.2)  69 (90.8)  1 | 0.275^b^ | 11 (14.3)  66 (85.7)  1 | 0 (0)  22 (100)  0 | 0.116^b^ |

Data are presented as number (%) from number of participants with available (non-missing) data. The "Total" group includes the total study population; the "Any STI" group includes participants who tested positive for at least one STI, including the co-infections; the "No STI" group includes participants who tested negative for all STIs; the “PID” group includes participants who were diagnosed with PID; and the “TOA + PID” group includes participants who were diagnosed with both TOA and PID. Either the ^a^Chi-square test or ^b^Fisher’s exact test was performed to indicate significant differences in the frequency of experienced symptoms and signs between STI status groups (any STI or no STI) and diagnosis groups (PID or TOA + PID). STI, sexually transmitted infection; PID, pelvic inflammatory disease; TOA, tubal-ovarian abscess. CRP, C-reactive protein.

|  | **n** | **Day 1** | **Day 3** | **Day 5** | **Day 7** | **Day 14** | **Day 28** | **Reduction^i^ (%)** |
| --- | --- | --- | --- | --- | --- | --- | --- | --- |
| Total | 71 | 23 (16-27)^d^ | 18 (13-22)^a^ | 13 (8-19) | 9 (5-14)^e^ | 6 (3-9)^c^ | 2 (0.5-6)^f^ | 91.3^h^ |
| Any STI | 16 | 24 (22-27)^a^ | 16 (9-25)^a^ | 11 (5-18.5) | 7 (2.5-11.5) | 4 (2-7)^a^ | 4 (1-10)^c^ | 83.3^d^ |
| No STI | 55 | 21.5 (15-27.5)^c^ | 18 (13-22) | 14 (9-20) | 10 (7-15)^e^ | 6 (3-9)^b^ | 2 (0-5)^d^ | 90.7^g^ |
| PID | 53 | 23 (17-27)^c^ | 18 (13-21) | 13 (9-19) | 9 (5-15)^c^ | 6 (3-9)^b^ | 2.5 (1-7.5)^e^ | 89.1^g^ |
| TOA + PID | 18 | 20 (14-27)^a^ | 19 (15-23)^a^ | 14 (7-21) | 10.5 (4-13)^b^ | 5 (3-8)^a^ | 2 (0-4)^b^ | 90.0^d^ |

**Table S2**. Combined symptom score stratified by STI status and diagnosis

Data are presented as median scores with interquartile range. Combined symptom score is calculated as the sum of eight symptoms: abdominal pain, shivering, dysuria, abnormal discharge, intermenstrual bleeding, malaise, nausea, and fatigue. The "Total" group includes the total study population; the "Any STI" group includes participants who tested positive for at least one STI, including the co-infections; the "No STI" group includes participants who tested negative for all STIs; the “PID” group includes participants who were diagnosed with PID; and the “TOA + PID” group includes participants who were diagnosed with both TOA and PID. ^a^One case missing. ^b^Two cases missing. ^c^Three cases missing. ^d^Four cases missing. ^e^Five cases missing. ^f^Seven cases missing. ^g^Nine cases missing. ^h^Thirteen cases missing. ^i^Percentage reduction in median symptom scores between days 1 and 28. STI, sexually transmitted infection; PID, pelvic inflammatory disease; TOA, tubal-ovarian abscess.

**Table S3**. Clinical outcomes based on self-reported symptom diary

|  | **Day 1**  **(n=72)** | **Day 3**  **(n=72)** | **Day 5**  **(n=72)** | **Day 7**  **(n=71)** | **Day 14**  **(n=72)** | **Day 28**  **(n=69)** | **Reduction^f^ (%)** |
| --- | --- | --- | --- | --- | --- | --- | --- |
| Abdominal pain | 5 (4-5) | 3 (2-4) | 2 (1-3) | 2 (1-3) | 1 (0-2) | 0 (0-1) | 100 |
| Shivers | 3 (0-4)^e^ | 0 (0-2) | 0 (0-1) | 0 (0-0)^a^ | 0 (0-0)^c^ | 0 (0-0)^b^ | 100 |
| Pain during urination | 1.5 (0-3)^b^ | 1 (0-2) | 0 (0-1) | 0 (0-1)^a^ | 0 (0-0) | 0 (0-0)^c^ | 100 |
| Unusual discharge | 1 (0-3)^b^ | 1 (0-3) | 1 (0-2) | 0 (0-2)^b^ | 0 (0-1) | 0 (0-1)^e^ | 100 |
| Intermenstrual bleeding | 0 (0-2)^b^ | 0 (0-2)^a^ | 0 (0-1.5) | 0 (0-1)^a^ | 0 (0-0) | 0 (0-0)^c^ | NA^g^ |
| Malaise | 4.5 (3-5) | 3 (3-4) | 2 (1-4) | 1 (0-3) | 0 (0-1)^a^ | 0 (0-0)^a^ | 100 |
| Nausea | 3 (1-5)^a^ | 3 (1-4)^a^ | 2 (0-4)^a^ | 0.5 (0-2)^a^ | 0 (0-2)^a^ | 0 (0-0)^a^ | 100 |
| Tiredness | 4 (4-5)^a^ | 4 (3-5) | 4 (2-5)^a^ | 3 (2-4)^a^ | 2 (1-4)^a^ | 1 (0-2) | 75.0 |
| Fever, n (%) | 39 (54.2) | 10 (14.1)^a^ | 4 (5.6) | 1 (1.4)^a^ | 0 (0) | 1 (1.5)^b^ | 97.2 |
| Vomiting, n (%) | 16 (22.2) | 9 (12.7)^a^ | 6 (8.5)^a^ | 4 (5.8)^b^ | 3 (4.2) | 0 (0)^b^ | 100 |

Data are presented as median scores with interquartile ranges, unless otherwise indicated. All scores are on a 0-5 Likert scale, except for fever and vomiting, which were recorded as binary (yes/no) outcomes. ^a^One case missing. ^b^Two cases missing. ^c^Three case missing. ^d^Four cases missing. ^e^Five cases missing. ^f^Percentage reduction in median symptom scores between days 1 and 28. ^g^Not applicable (NA), as median scores were zero.

**Table S4**. Clinical symptom scores of participants with <70% reduction in symptom scores

| **Participant id** | **Day 1^a^** | **Day 28^a^** | **Reduction (%)^b^** |
| --- | --- | --- | --- |
| 6 | 15 | 9 | 40.0 |
| 11 | 17 | 7 | 58.8 |
| 23 | 40 | 13 | 67.5 |
| 27 | 12 | 8 | 33.3 |
| 30 | 13 | 8 | 38.5 |
| 32 | 20 | 9 | 55.0 |
| 46 | 34 | 15 | 55.9 |
| 60 | 22 | 10 | 54.5 |
| 63 | 28 | 16 | 42.9 |
| 67 | 35 | 12 | 65.7 |
| 71 | 23 | 10 | 56.5 |
| 73 | 18 | 6 | 55.7 |
| 88 | 25 | 8 | 68.0 |
| 89 | 6 | 4 | 33.3 |

^a^Combined symptom score is calculated as the sum of eight symptoms: abdominal pain, shivering, dysuria, abnormal discharge, intermenstrual bleeding, malaise, nausea, and fatigue. ^b^Percentage reduction in median symptom scores between days 1 and 28.
